# Phase III trial comparing paclitaxel poliglumex *vs* docetaxel in the second-line treatment of non-small-cell lung cancer

**DOI:** 10.1038/sj.bjc.6604372

**Published:** 2008-05-13

**Authors:** L Paz-Ares, H Ross, M O'Brien, A Riviere, U Gatzemeier, J Von Pawel, E Kaukel, L Freitag, W Digel, H Bischoff, R García-Campelo, N Iannotti, P Reiterer, I Bover, J Prendiville, A J Eisenfeld, F B Oldham, B Bandstra, J W Singer, P Bonomi

**Affiliations:** 1 Hospital Universitario 12 de Octubre, Servicio de Oncología Médica, Madrid 28041, Spain; 2 Earle A. Chiles Research Institute, Portland, OR 97213, USA; 3 Royal Marsden Hospital, London SW3 6JJ, UK; 4 Centre François Baclesse, Centre de Lutte Contre le Cancer de Caen, Caen 14076, France; 5 Krankenhaus Grosshansdorf, Grosshansdorf 22927, Germany; 6 Asklepios-Fachkliniken Munchen Gauting, Gauting 82131, Germany; 7 Allgemeines Krankenhaus Harburg, Hamburg 21075, Germany; 8 Lungenklinik Hemer, Hemer 58675, Germany; 9 Universitatsklinikum Freiburg, Freiburg 79106, Germany; 10 Department of Radiology, Thoraxklinik Heidelberg GmbH, Heidelberg 69126, Germany; 11 Juan Canalejo University Hospital, Servicio de Oncología Médica, La Coruña 15006, Spain; 12 Hem-Onc Associates of The Treasure Coast, Port St. Lucie, FL 34952, USA; 13 Masarykova Nemocnice, Ústí nad Labem 401 13, Czech Republic; 14 Hospital San Llàtzer, Palma de Mallorca 07198, Spain; 15 Guy's Hospital, Medical Oncology and Breast Unit, London SE1 9RT, UK; 16 Cell Therapeutics Inc., Seattle, WA 98119, USA; 17 Rush University Medical Center, 1725 West Harrison Street, Chicago, IL 60612, USA

**Keywords:** lung cancer, poliglumex, docetaxel

## Abstract

Paclitaxel poliglumex (PPX), a macromolecule drug conjugate linking paclitaxel to polyglutamic acid, reduces systemic exposure to peak concentrations of free paclitaxel. Patients with non-small-cell lung cancer (NSCLC) who had received one prior platinum-based chemotherapy received 175 or 210 mg m^−2^ PPX or 75 mg m^−2^ docetaxel. The study enrolled 849 previously treated NSCLC patients with advanced disease. Median survival (6.9 months in both arms, hazard ratio=1.09, *P*=0.257), 1-year survival (PPX=25%, docetaxel=29%, *P*=0.134), and time to progression (PPX=2 months, docetaxel=2.6 months, *P*=0.075) were similar between treatment arms. Paclitaxel poliglumex was associated with significantly less grade 3 or 4 neutropenia (*P*<0.001) and febrile neutropenia (*P*=0.006). Grade 3 or 4 neuropathy (*P*<0.001) was more common in the PPX arm. Patients receiving PPX had less alopecia and did not receive routine premedications. More patients discontinued due to adverse events in the PPX arm compared to the docetaxel arm (34 *vs* 16%, *P*<0.001). Paclitaxel poliglumex and docetaxel produced similar survival results but had different toxicity profiles. Compared with docetaxel, PPX had less febrile neutropenia and less alopecia, shorter infusion times, and elimination of routine use of medications to prevent hypersensitivity reactions. Paclitaxel poliglumex at a dose of 210 mg m^−2^ resulted in increased neurotoxicity compared with docetaxel.

Lung cancer is the most common cancer and one of the most lethal cancers. In the United States, an estimated 213 380 new lung cancer cases and 160 390 lung cancer deaths were expected in 2007 (Jemal *et al*, 2007). For patients who present with advanced-stage disease (IIIb or IV), platinum-based multi-agent chemotherapy modestly improves survival compared with best supportive care or single-agent therapy (Pfister *et al*, 2004). However, nearly all patients relapse, and only 10–20% survive 2 years. Three agents are currently approved for second-line therapy in advanced non-small-cell lung cancer (NSCLC). Docetaxel and erlotinib were approved on the basis of improved survival compared with best supportive care (Shepherd *et al*, 2000, 2005). Pemetrexed received approval as second-line therapy due to its similarity in survival and response rates with lower toxicity than that of docetaxel, although statistical noninferiority was not achieved (Hanna *et al*, 2004). Despite response rates of approximately 10%, second-line treatment improves survival by approximately 2 months compared with best supportive care.

Owing to the palliative nature of second-line therapy in NSCLC and its relatively modest effect on survival, minimising the toxicity of therapy is an important consideration. Additional effective therapies that achieve that goal are needed.

Paclitaxel poliglumex (PPX) is a macromolecular polymer–drug conjugate that links paclitaxel to a biodegradable polymeric backbone consisting of L-glutamic acid residues. Because the conjugation site is through the 2′ hydroxyl of paclitaxel, a site crucial for tubulin binding, conjugated paclitaxel does not interact with *β*-tubulin and is biologically inactive (Singer *et al*, 2005). Paclitaxel poliglumex is relatively stable in circulation; the area under the curve (AUC) of unconjugated paclitaxel is 1–2% of the AUC of conjugated paclitaxel. Clinical plasma pharmacokinetics of PPX show a biphasic decline with a prolonged distribution phase and an elimination phase with a long terminal half-life (Bernareggi *et al*, 2005). The total systemic exposure to unconjugated paclitaxel is similar after administration of equivalent doses of PPX and standard paclitaxel; however, the *C*_max_ values for paclitaxel are significantly lower in patients treated with PPX. (Bernareggi *et al*, 2005). The single-cycle, maximum tolerated dose of PPX in phase Ia study was 233 mg m^−2^ every 3 weeks, with neutropenia being the dose-limiting toxicity (Boddy *et al*, 2005). In the phase Ib portion of the trial, in which CT-2103 was administered every 2 weeks, the maximum tolerated dose was 177 mg m^−2^, with neuropathy being the dose-limiting side effect. Neither study was able to address multi-cycle toxicities. A dose of 210 mg m^−2^ was chosen for the current study in view of the expectation that most patients will have had prior paclitaxel therapy and a potential for cumulative neuropathy with prolonged administration of CT-2103.

Macromolecules such as PPX passively accumulate in tumour tissues by taking advantage of the hyperpermeable tumour vasculature and reduced lymphatic clearance. This phenomenon is known as the enhanced permeation and retention (EPR) effect, which results in a 10- to 100-fold increase in intratumoral drug concentrations when compared with an equivalent dose of the drug given conventionally (Matsumura and Maeda, 1986; Greish *et al*, 2003). To take advantage of the EPR effect, macromolecules have to remain in circulation for at least 6 h (Matsumura and Maeda, 1986). The prolonged circulation time of PPX facilitates tumour accumulation through the EPR effect, as has been demonstrated in animal models (Li *et al*, 2000). The release of paclitaxel from the polymeric backbone is dependent on lysosomal proteases, particularly cathepsin B (Shaffer *et al*, 2007). In malignant tumours and premalignant lesions, increased cathepsin B mRNA expression is associated with elevated cathepsin B protein levels and activity and correlates with tumour invasion (Podgorski and Sloane, 2003).

In a phase II trial of 28 patients who were either elderly, had performance status (PS) 2, or both with treatment-naïve advanced NSCLC, PPX at a dose of 175 mg m^−2^ every 3 weeks yielded a median survival of 8.1 months in a PS 0–1 population and 5.4 months in a PS 2 cohort (Richards *et al*, 2005). This dose was well tolerated with a median of three cycles administered. The disease control rate was 71% (2 partial remissions and 15 patients with stable disease).

Given the enhanced efficacy of PPX in preclinical models, its activity in phase II, and its tolerability, the present trial was initiated to compare survival in NSCLC patients treated with PPX to that of docetaxel as second-line therapy in patients who had previously received a platinate combination.

## MATERIALS AND METHODS

The study was approved by the local research ethics committees. All patients gave written informed consent.

### Study design

STELLAR 2 was an open-label, phase III study comparing docetaxel with PPX. Paclitaxel poliglumex was administered as a 10- to 20-min infusion at 210 mg m^−2^ for advanced disease NSCLC patients with Eastern Cooperative Oncology Group PS 0 or 1 and at 175 mg m^−2^ for patients with PS 2. The dose was reduced for PS 2 patients after the trial had started based on the results from an ongoing study in PS 2 patients. In that study, the data monitoring committee noted an increased incidence of death resulting from neutropenia in 96 patients treated with 235 mg m^−2^ PPX. As a result of these observations, the dose was reduced to 175 mg m^−2^ PPX (O’Brien *et al*, in press). Docetaxel was administered as a 1-h intravenous (i.v.) infusion at 75 mg m^−2^. In both study arms, patients received i.v. treatment every 3 weeks. Patients in the docetaxel arm received routine hypersensitivity reaction (HSR) prophylaxis including corticosteroids (e.g. dexamethasone 20 mg i.v.), histamine 2 receptor blockers (e.g. cimetidine 300 mg i.v.), and antihistamines (e.g. diphenhydramine 50 mg i.v.). These agents were administered just before chemotherapy. Patients in the PPX arm received *no* standard HSR prophylaxis. Patients were treated until disease progression, intolerable toxicity, patient withdrawal of consent, or investigator decision to stop treatment.

All concurrent medications were recorded. Anti-emetic prophylaxis was permitted. Granulocyte colony-stimulating factor (G-CSF) and granulocyte–macrophage colony-stimulating factor were administered according to American Society of Clinical Oncology guidelines.

Patients were stratified based on stage (IV *vs* other), PS (0 or 1 *vs* 2), start of front-line (platinum-based) chemotherapy (<16 weeks from randomisation *vs* ⩾16 weeks from randomisation), gender, and prior taxane therapy (yes *vs* no).

### End points

The primary end point of this study was the comparison of overall survival of patients treated with PPX to that of docetaxel. No patients were censored regardless of the apparent cause of death. Secondary objectives included response rate, time to progression (TTP), safety, and quality of life. Response status was established by response evaluation criteria in solid tumours (RECIST) (Therasse *et al*, 2000). Computed tomography (CT) or other imaging techniques were used to assess patients during the third week of every other cycle. For patients who completed therapy and had no evidence of disease progression, re-evaluation of indicator lesions was obtained every 8 weeks until documentation of disease progression or alternative therapy.

Safety data were collected on all patients. In addition, disease-related symptoms were measured by the functional assessment of cancer therapy-lung cancer symptom (FACT-LCS) scale at baseline and within 3 days of each treatment administration.

### Eligibility

All patients enrolled in this study had histologically or cytologically confirmed advanced NSCLC and had been treated with a single platinum-based systemic therapy. Patients who received radiation sensitising doses of platinum-based chemotherapy with concurrent chest radiation were not eligible. Patients who received full doses of adjuvant chemotherapy were eligible. Patients were ⩾18 years with adequate end organ indices, including baseline absolute neutrophil count (ANC) ⩾1500 *μ*l^−1^, platelet count ⩾100 000 *μ*l^−1^, adequate renal function defined as creatinine ⩽1.5 times the upper limit of normal, bilirubin >1.0 times the upper limit of normal, transaminases ⩽2.5 times the upper limit of normal (⩽5 times the upper limit if hepatic metastases were documented), and alkaline phosphatase ⩽2.5 times the upper limit (unless documented bone metastases were present). Patients with known brain metastases were required to have stable disease after standard antitumour treatment (e.g. whole brain radiation, stereotactic radiation, or surgical resection) and be either off corticosteroid treatment or on tapering doses. Those who had undergone surgery had to be fully recovered. Patients with reproductive potential were required to commit to adequate contraception.

Exclusion criteria included evidence of small cell, carcinoid, or mixed small cell/NSCLC histologies; no previous treatment with a platinum regimen for NSCLC; other concurrent, active primary malignancies requiring treatment with the exception of carcinoma *in situ* of the uterine cervix or nonmelanomatous skin cancer; baseline grade ⩾2 neuropathy; clinically significant infection; exposure to other investigational agents within 4 weeks of study entry; unstable medical conditions, including myocardial infarction within the prior 6 months, inadequately treated chronic obstructive pulmonary disease, or significant arrhythmias. All patients were required to sign informed consents.

### Criteria for removal from study

Patients were treated until documentation of disease progression clinically or on CT imaging or in the event of intolerable toxicities, including persistent grade ⩾3 nonhaematologic toxicities, grade 4 HSR, grade 3 HSR despite adequate prophylaxis, or other toxicities precluding study continuation. Other criteria for treatment discontinuation included withdrawal of consent, individual physician discretion for reasons unrelated to toxicities, and violation of study protocol, including patient noncompliance.

### Dosing and dose modifications

The doses of study agents were reduced for the following conditions: (1) febrile neutropenia at any time; (2) grade 4 neutropenia lasting >7 days; (3) failure to recover to ANC of 1500 *μ*l^−1^ by day 22; (4) platelet count >20 000 or <50 000 with associated bleeding; (5) grade ⩾2 neuropathy; (6) any other attributable grade 3 or 4 nonhaematologic toxicity, with the exception of manageable nausea and vomiting or HSR; and (7) dose delays due to drug-related toxicity.

For PPX, the first onset of dose-limiting toxicity mandated a dose reduction to 175 mg m^−2^ for patients whose initial dose was 210 and to 135 mg m^−2^ for initial doses of 175 mg m^−2^; second dose reductions mandated a decrease to 135 or 90 mg m^−2^, respectively. Once a dose reduction was instituted, doses were not re-escalated. Patients who experienced grade ⩽3 HSR during or following treatment were allowed to continue treatment at investigator's discretion, but were required to receive standard premedication in accordance with institutional guidelines.

For docetaxel, the first onset of dose-limiting toxicity mandated a dose reduction to 75 mg m^−2^; second dose reductions mandated a decrease to 55 mg m^−2^. Once a dose reduction was instituted, doses were not re-escalated. Patients who experienced grade ⩽3 HSR during or following treatment were allowed to continue treatment at investigator's discretion, but were required to receive standard premedication in accordance with institutional guidelines.

### Efficacy parameters and statistical considerations

Overall survival was defined as the interval between randomisation and death from any cause. Patients remaining alive, including those lost to follow-up, were censored at the date of last contact. Nonstratified log rank testing was used for the formal primary comparison of survival. This study targeted accrual of 840 evaluable patients, which guaranteed 80% power and 0.05 type I error to show a 1.5-month improvement (30% increase) in median survival from baseline of 6 to 7.5 months. The study was slated to accrue over 18 months with an additional 6 months of follow-up. A secondary, noninferiority analysis of overall survival was also performed using the fraction retention method described by Rothmann *et al* (2003). In addition, secondary analyses comparing each treatment arm were conducted using Cox regression models, which included covariates that reflected prognostic factors associated with survival in patients with NSCLC.

Response was assessed according to RECIST criteria. Disease control was determined by the percentage of patients alive without disease progression for at least 12 weeks. All randomised patients were included in these comparisons using Fisher's exact test.

Time to progression was defined as the time interval between randomisation and the first observation of disease progression due to any cause. Primary analysis of TTP was made using an unstratified log rank test. Secondary analyses of TTP were performed using Cox regression models with covariates used in the secondary analysis of survival.

Safety variables were summarised by descriptive statistics for patients who received any study treatment. All toxicities were graded according to the National Cancer Institutes Common Toxicity Criteria, version 2. Toxicities were compared between the treatment arms using Fisher's exact test.

### Quality of life

Disease-related symptoms were measured by the FACT-LCS scale, a validated, 5-point Likert-type scale ranging from 0 (not at all) to 4 (very much). The total LCS score ranged from 0 to 28, with higher scores indicative of fewer symptoms. Fisher's exact test for equal proportion of patients achieving at least a 2-point increase in FACT-LCS score from baseline to week 3 was performed in the overall sample and by each baseline covariate strata. Patients with a missing FACT-LCS score at week 3 were classified as having a <2-point increase in the primary analysis data, but classified as missing and excluded from the supplemental analysis.

## RESULTS

### Disposition of patients

A total of 849 patients were randomised to receive either PPX (*n*=427) or docetaxel (*n*=422). Five patients (two patients had progressive disease, one patient requested withdrawal, one patient did not comply with the protocol, and no reason was given for one patient) in the PPX treatment arm and six patients (one patient died of pulmonary embolism before receiving study drug, two patients had progressive disease, one patient requested withdrawal, the physician requested withdrawal not related to toxicity for one patient, and one patient did not comply with the protocol) in the docetaxel arm were randomised but did not receive study drug. The first patient was randomised on 18 October 2002 and the last patient was enrolled on 13 August 2004.

Demographic breakdown is included in Table 1. Both arms were well balanced with regard to baseline characteristics: 72% of patients were male, 92% were Caucasian, 81% had stage IV disease, and 29% of patients had received prior taxanes. The median age was 61 years in the PPX arm and 62 in the docetaxel arm. The majority of patients (57%) came from western Europe and Canada; 34% came from the United States.

The most frequent reasons for stopping treatment were progressive disease (57% in the PPX arm compared with 63% in the docetaxel arm, *P*=0.107) and adverse events (34% in the PPX arm compared with 16% in the docetaxel arm, *P*<0.001). Additionally, 4% of patients in the PPX arm withdrew consent compared with 8% in the docetaxel arm (*P*=0.011).

#### Efficacy summary

**Figure 1:**
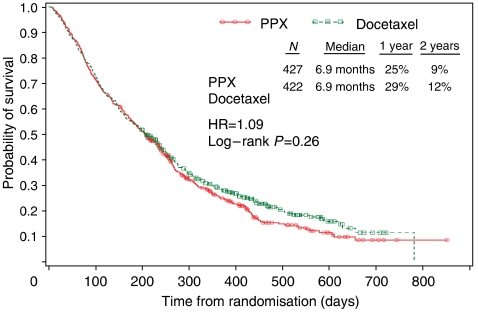
Kaplan–Meier plot of overall survival (intent-to-treat data set).

Median overall survival was 6.9 months in both arms of the study (*P*=0.26) (Table 3). One-year survival rates were 25% in the PPX arm and 29% in the docetaxel arm (*P*=0.134). The 2-year survival rates were higher in the docetaxel arm (12 compared with the PPX arm (9%)). These differences were not statistically significant (*P*=0.195). Survival curves are shown in Figure 1.

Noninferiority, defined as retention of ⩾90% of docetaxel effect, was not observed between the two arms (hazard ratio (HR)=1.09; 95% confidence interval (CI)=0.94–1.27). In subsequent analyses, a method that uses historic effect size estimates of placebo-controlled trials to adjust the HR observed when working with a nonplacebo control was implemented. The results from this method yielded an HR of 0.61 (95% CI=0.38, 0.98), indicating that PPX is an active agent when indirectly compared with placebo.

### Response and progression data

The overall response rate for the PPX arm was 8%, with no complete responses (CRs) (Table 3). The overall response rate for the docetaxel arm was 12%, with two CRs. Disease control, defined as absence of progression during the first 12 weeks, occurred in 40% in the PPX arm compared with 45% in the docetaxel arm.

**Figure 2:**
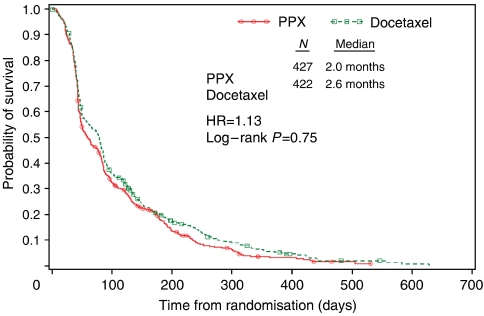
Kaplan–Meier plot of time to disease progression (intent-to-treat data set). HR, hazard ratio; PPX, paclitaxel poliglumex.

There was no significant difference in median TTP: 2.0 months in the PPX arm compared with 2.6 months in the docetaxel arm (HR=1.13; log rank *P*=0.075). Time to progression curves are shown in Figure 2. There was no difference in subsequent therapies: 11% of those enrolled in the PPX arm went on to radiation therapy compared with 13% in the docetaxel arm. In both arms, 57% received additional chemotherapy, but no specific agents predominated.

### Quality of life

The primary FACT-LCS analysis consisted of 767 patients (PPX: *n*=379; docetaxel: *n*=388). There was no difference between the two treatment groups in the proportion of subjects achieving at least a 2-point increase in FACT-LCS score from baseline to cycle 3 (*P*=0.329). Both treatment groups reported similar proportions of FACT-LCS scale score and item score changes from baseline over time. During the study period, 41% of patients treated with docetaxel achieved at least a 2-point improvement in FACT-LCS score from baseline compared with 34% of patients treated with PPX.

### Toxicity profile

#### Drug delivery

The median number of cycles received was two in the PPX arm and three in the docetaxel arm. More patients received ⩾6 cycles in the docetaxel arm (*P*<0.001). In aggregate, patients received >90% of mean expected dose during the second and subsequent cycles.

### Relative toxicities

Patients enrolled in the docetaxel arm were significantly more likely to experience grade 3 or 4 neutropenia (37 *vs* 14%, *P*<0.001) and febrile neutropenia (6 *vs* 2%, *P*=0.006) (Table 2). Alopecia also occurred more frequently in the docetaxel arm (43 *vs* 14%, *P*<0.001). Patients enrolled in the PPX arm were significantly more likely to experience grade 3 or 4 HSR (3 *vs* <1%, *P*=0.007) and neuropathy (19 *vs* 3%, *P*<0.001). Neuropathy of all grades occurred in 50% of patients in the PPX arm and 30% of patients in the docetaxel arm. Severe neuropathy (common toxicity criteria grade 3 or 4) was observed in 19% of patients in the PPX arm and 3% of patients in the docetaxel arm. Of note, only grade 3 events were seen in the docetaxel arm. There was a general trend towards an increasing incidence of severe neuropathy by cycle in the PPX arm through cycle 4. The mean cumulative dose of PPX at the first event of neuropathy was 532.9 mg m^−2^. A similar pattern was not observed in the docetaxel arm.

The incidence of febrile neutropenia was 2% in the PPX arm compared with 6% in the docetaxel arm (*P*=0.002). The use of supportive care, including transfusions, erythropoietin, and G-CSF, was lower in the PPX treatment arm. Grade 3 or 4 infections occurred more frequently in the docetaxel arm (11%) than in the PPX arm (7%).

Twelve per cent of patients in the PPX arm and 16% of patients in the docetaxel arm died within 30 days of treatment, but only 2% of these deaths were attributable to study drugs, 9% appeared to be disease related, and 3% were due to comorbidities.

The incidence of HSR was 5% for the PPX arm, *without* routine HSR prophylaxis, compared with 3% for the docetaxel arm, *with* HSR prophylaxis.

## DISCUSSION

Paclitaxel poliglumex and docetaxel produced similar results for TTP and overall survival in one of the largest phase III studies to date evaluating second-line chemotherapy in NSCLC. The TTP and survival results in the current trial are similar to results reported for docetaxel, pemetrexed, and erlotinib observed in smaller randomised studies (Fossella *et al*, 2000; Shepherd *et al*, 2000; Hanna *et al*, 2004) (Table 3). Collectively, these data suggest that second-line therapy with an active agent has a modest survival advantage over supportive care alone and that the effects of docetaxel, PPX, pemetrexed, and erlotinib are similar.

Patients treated with PPX received a median of two cycles of therapy *vs* three cycles in the docetaxel arm with more patients withdrawing from PPX for adverse events and more patients in the docetaxel arm withdrawing for progressive disease. The dose of 210 mg m^−2^ PPX used in this study may have been higher than optimal and may have been responsible for the relatively high withdrawal rate. Nevertheless, patients treated with PPX had significantly less neutropenia and febrile neutropenia and required less growth factor and transfusion support. However, they did experience more grade 3 neuropathy (19 *vs* 3%), a common cause for discontinuation. A general trend towards increasing incidence by cycle was seen in the PPX arm. Additional experience in patients with other diseases suggests that the optimal dose for repeated cycles of PPX is 175 mg m^−2^ with early dose reduction for development of even grade 1 neuropathy. In this study, the dose was reduced only when persistent grade 2 neuropathy had developed. Studies with paclitaxel have consistently failed to demonstrate a dose–response relationship. In contrast, in a phase III study of single-agent PPX at 175 mg m^−2^, grade 3 neuropathy occurred in 4% of patients despite administration of a median of four cycles of therapy (O’Brien *et al*, in press). In that study in PS 2 patients, PPX was not inferior to single-agent therapy with either gemcitabine or vinorelbine (median survival=220 *vs* 198 days, respectively; HR=0.95) and produced fewer grade 3 or 4 toxicities.

Paclitaxel poliglumex has advantages over docetaxel in ease of administration, requiring a 10- to 20-min peripheral vein infusion without routine premedications and a low incidence of neutropenic fever or infections. An additional advantage is the decreased rate of alopecia (43 *vs* 14%) due to reduced systemic exposure to high levels of free paclitaxel. Despite prior taxane exposure in 30% of patients treated with PPX, the incidence of HSR was only 5% (3% grade 3 or 4) compared with 3% (<1% grade 3 or 4) for docetaxel.

Preclinical and clinical studies suggest an interaction between PPX and oestrogen (Ross *et al*, 2006). A clinical trial in women with oestradiol levels >30 pg ml^−1^ is being conducted to test the hypothesis that women with normal oestradiol levels who are treated with PPX and carboplatin will have improved survival compared to women treated with paclitaxel and carboplatin.

### Final conclusions

Paclitaxel poliglumex produces similar survival to docetaxel as second-line treatment in NSCLC with less febrile neutropenia and alopecia and greater ease of administration. The higher incidence of neuropathy can likely be reduced by lowering the starting dose to 175 mg m^−2^ and using early dose reduction for development of even grade 1 neuropathy. Additional studies in patients undergoing second-line therapy are needed to validate this.

## Figures and Tables

**Table 1:** Demographic and baseline characteristics

	**PPX (*n*=427)**	**Docetaxel (*n*=422)**
*Gender*		
Male	308 (72.1%)	302 (71.6%)
Female	119 (27.9%)	120 (28.4%)
		
*Race*		
Caucasian	397 (93.0%)	381 (90.3%)
Black	19 (4.4%)	21 (5.0%)
Asian	5 (1.2%)	5 (1.2%)
Hispanic	4 (0.9%)	11 (2.6%)
Other	1 (0.2%)	4 (0.9%)
Unknown	1 ( 0.2%)	0 ( 0%)
		
*Age at randomisation*		
*N*	427	422
Mean (s.d.)	61.3 (9.81)	62.1 (9.72)
Median (range)	62.0 (30–87)	63.0 (34–85)
		
*Geographic location*		
United States	150 (35.1%)	140 (33.2%)
Western Europe and Canada	240 (56.2%)	245 (58.1%)
Other	37 (8.7%)	37 (8.8%)
		
*ECOG performance rating*		
0	93 (22%)	80 (19%)
1	277 (65%)	280 (66%)
2	57 (13%)	62 (15%)
		
*Disease stage*		
IV	342 (80%)	343 (81%)
Other	84 (20%)	75 (18%)
Unknown	1 (<1%)	4 (<1%)
		
*Time since start of first-line chemotherapy*		
<16 weeks	132 (31%)	133 (32%)
⩾16 weeks	295 (69%)	289 (68%)
		
*Prior taxane use*		
Yes	127 (30%)	123 (29%)
No	286 (67%)	287 (68%)
Unknown	14 (3%)	12 (3%)

ECOG=Eastern Cooperative Oncology Group; PPX=paclitaxel poliglumex.

**Table 2:** Number (%) of patients with select adverse events

	**PPX (*n*=422)**		**Docetaxel (*n*=416)**		
**Toxicity**	**All**	**Grade 3 or 4**	**All**	**Grade 3 or 4**	
Anaemia NOS	73 (17)	19 (5)	109 (26)	17 (4)	0.002
Neutropenia	87 (21)	58 (14)	183 (44)	152 (37)	<0.001
Leukopenia NOS	48 (11)	28 (7)	26 (6)	8 (2)	0.004
Thrombocytopaenia	31 (7)	10 (2)	18 (4)	3 (<1)	0.077
Febrile neutropenia	8 (2)	8 (2)	25 (6)	23 (6)	0.002
Anorexia	96 (23)	12 (3)	72 (17)	27 (6)	0.057
Dehydration	24 (6)	10 (2)	23 (6)	8 (2)	1.000
Neuropathy NOS	211 (50)	81 (19)	123 (30)	14 (3)	<0.001
Dyspnoea NOS	99 (23)	50 (12)	105 (25)	51 (12)	0.574
Nausea	140 (33)	14 (4)	139 (33)	8 (2)	1.000
Vomiting NOS	82 (19)	9 (2)	72 (17)	17 (4)	0.476
Diarrhoea NOS	67 (16)	6 (1)	108 (26)	11 (3)	<0.001
Stomatitis/mucositis	32 (8)	2 (<1)	93 (22)	10 (2)	<0.001
Arthralgia	51 (12)	2 (<1)	43 (10)	4 (<1)	0.445
Fatigue	115 (27)	24 (6)	148 (36)	35 (8)	0.011
Asthenia	68 (16)	21 (5)	95 (23)	24 (6)	0.015
Weight loss	63 (13)	3 (<1)	41 (10)	11 (3)	0.230
Alopecia	38 (9)	NA	134 (32)	NA	<0.001

NA=not applicable; NOS=not otherwise specified; PPX-paclitaxel poliglumex.

**Table 3:** Outcomes of second-line single-agent treatment of NSCLC

**Reference**	**Drug**	***N***	**Median survival (months)**	**1-year survival (%)**	**TTP (months)**
Fossella *et al*.	Docetaxel	125	5.7	32	2.0
Hanna *et al*.	Docetaxel	288	7.9	30	3.5
PGT302	Docetaxel	422	6.9	29	2.6
Hanna *et al*.	Pemetrexed	283	8.3	30	3.4
Shepherd *et al*.	Erlotinib	41	6.7	NA	2.2
PGT302	PPX	427	6.9	25	2.0

NA=not applicable; NSCLC=non-small-cell lung cancer; PPX=paclitaxel poliglumex; TTP=time to progression.
